# Unplanned pregnancy in Brazil: national study in eight university hospitals

**DOI:** 10.11606/s1518-8787.2023057004449

**Published:** 2023-06-02

**Authors:** Tainá Vieira Nilson, Angélica Amorim Amato, Ceres Nunes Resende, Walquíria Quida Salles Pereira Primo, Roseli Mieko Yamamoto Nomura, Maria Laura Costa, Maria Lúcia Opperman, Marianna Brock, Alberto Trapani, Lia Cruz Vaz da Costa Damasio, Nadia Reis, Vera Borges, Ana Cristina Araújo, Rodrigo Ruano, Alberto Carlos Moreno Zaconeta

**Affiliations:** I Universidade de Brasília Hospital Universitário de Brasília Brasília DF Brasil Universidade de Brasília. Hospital Universitário de Brasília. Brasília, DF, Brasil; II Universidade de Brasília Faculdade de Ciências da Saúde Departamento de Ciências Farmacêuticas Brasília DF Brasil Universidade de Brasília. Faculdade de Ciências da Saúde. Departamento de Ciências Farmacêuticas. Brasília, DF, Brasil; III Universidade de Brasília Faculdade de Medicina Área de Ginecologia e Obstetrícia Brasília DF Brasil Universidade de Brasília. Faculdade de Medicina. Área de Ginecologia e Obstetrícia. Brasília, DF, Brasil; IV Universidade Federal de São Paulo Escola Paulista de Medicina Departamento de Obstetrícia São Paulo SP Brasil Universidade Federal de São Paulo. Escola Paulista de Medicina. Departamento de Obstetrícia. São Paulo, SP, Brasil; V Universidade Estadual de Campinas Departamento de Ginecologia e Obstetrícia Campinas SP Brasil Universidade Estadual de Campinas. Departamento de Ginecologia e Obstetrícia. Campinas, SP, Brasil; VI Universidade Federal do Rio Grande do Sul Faculdade de Medicina Unidade de Ginecologia e Obstetrícia Porto Alegre RS Brasil Universidade Federal do Rio Grande do Sul. Faculdade de Medicina. Unidade de Ginecologia e Obstetrícia. Porto Alegre, RS, Brasil; VII Universidade Federal do Amazonas Departamento de Obstetrícia e Ginecologia Manaus AM Brasil Universidade Federal do Amazonas. Departamento de Obstetrícia e Ginecologia. Manaus, AM, Brasil; VIII Universidade Federal de Santa Catarina Hospital Universitário Polydoro Ernani de São Thiago Unidade de Saúde da Mulher Florianópolis SC Brasil Universidade Federal de Santa Catarina. Hospital Universitário Polydoro Ernani de São Thiago. Unidade de Saúde da Mulher. Florianópolis, SC, Brasil; IX Universidade Federal do Piauí Departamento de Ginecologia e Obstetrícia Teresina PI Brasil Universidade Federal do Piauí. Departamento de Ginecologia e Obstetrícia. Teresina, PI, Brasil; X Universidade Federal de Mato Grosso do Sul Hospital Universitário Maria Aparecida Pedrossian Unidade de Ginecologia e Obstetrícia Campo Grande MS Brasil Universidade Federal de Mato Grosso do Sul. Hospital Universitário Maria Aparecida Pedrossian. Unidade de Ginecologia e Obstetrícia. Campo Grande, MS, Brasil; XI Universidade Estadual Paulista Escola de Medicina de Botucatu Departamento de Ginecologia e Obstetrícia Botucatu SP Brasil Universidade Estadual Paulista. Escola de Medicina de Botucatu. Departamento de Ginecologia e Obstetrícia. Botucatu, SP, Brasil; XII Universidade Federal do Rio Grande do Norte Maternidade Januário Cicco Departamento de Ginecologia e Obstetrícia Natal RN Brasil Universidade Federal do Rio Grande do Norte. Maternidade Januário Cicco. Departamento de Ginecologia e Obstetrícia. Natal, RN, Brasil; XIII Mayo Clinic College of Medicine Department of Obstetrics and Gynecology Maternal-Fetal Medicine Division Rochester USA Mayo Clinic College of Medicine. Department of Obstetrics and Gynecology. Maternal-Fetal Medicine Division. Rochester, USA

**Keywords:** Unplanned Pregnancy, Family Development Planning, Hormonal Contraception, Reproductive Rights

## Abstract

**OBJECTIVE:**

To estimate the prevalence of unplanned pregnancy in eight public university hospitals, distributed in the five regions that make up Brazil.

**METHODS:**

A secondary analysis of a national multicenter cross-sectional study, carried out in eight public university hospitals between June 1 and August 31, 2020, in Brazil. Convenience sample including women who gave birth within sixty consecutive days and met the following criteria: over 18 years old; gestational age over 36 weeks at delivery; with a single and live newborn, without malformations.

**RESULTS:**

Sample composed of 1,120 postpartum women, of whom 756 (67.5%) declared that the pregnancy had not been planned. The median prevalence of unplanned pregnancy was 59.7%. The prevalence of unplanned pregnancy across hospitals differed significantly: Campinas (54.8%), Porto Alegre (58.2%), Florianópolis (59%), Teresina (61.2%), Brasília (64.3%), São Paulo (64.6%), Campo Grande (73.9%) and Manaus (95.3%) (p < 0.001). Factors significantly associated with unplanned pregnancy were maternal age, black color, lower family income, greater number of children, greater number of people living in household, and not having a partner.

**CONCLUSION:**

In the studied sample, about two thirds of the pregnancies were declared as unplanned. The prevalence of unplanned pregnancies was related to social and demographic factors and varied significantly across the university hospitals evaluated.

## INTRODUCTION

The reduction in family size due to postponed parenthood and lower birth rates is a global trend and suggests greater access to efficient contraceptive methods^1^ . However, recent data indicate that 48% of the pregnancies that occurred in the world in the last five years were unplanned, which represents 121 million cases per year or a global annual rate of 64 unplanned pregnancies (UP) for every thousand women between 15 and 49 years old^1^ . The annual rate of unplanned pregnancies per thousand women of reproductive age is inversely proportional to each country’s socioeconomic development level, and is 34 in developed countries, 66 in countries with medium development index, and 93 in those with a low index^1^ . Thus, countries with a low development index concentrate the highest UP rates.

In Brazil, a survey carried out in the South region investigated all births that occurred in a municipality throughout 2007 and found an unplanned pregnancy rate of 65% in the 2,500 women interviewed^2^ . A similar result was reported in a study carried out in 2010, which evaluated data from more than five thousand women in a capital city in the Northeast region and found a UP prevalence of 68%^3^ . In that same year, a questionnaire applied in all maternity hospitals in the city of Ribeirão Preto, in the Southeast region of the country, found a UP prevalence of 54% in the 7,500 women interviewed^4^ . Of greater scope, the study “Birth in Brazil”, which evaluated 24,000 women who gave birth between 2011 and 2012, showed that 55% of pregnancies were unplanned and that there were important differences in access to perinatal health in different regions of Brazil^4^ . Data from all these studies convergently indicated that the group of women with unplanned pregnancies had a high degree of social vulnerability^2 , 4 - 6^ .

A decade after the last survey, we had the opportunity to explore the prevalence of unplanned pregnancies in maternity wards in different regions of the country. This study was designed to determine the frequency of UP in the maternity wards of eight public university hospitals distributed throughout Brazil’s five geographic regions.

## METHODS

### Study Design and Sample Composition

This is a secondary data analysis of a larger study^7^ , whose multicenter cross-sectional design allowed the collection of data in cities in the five regions of Brazil (North, Northeast, Midwest, Southeast and South). Of the ten centers that participated in the larger study, two did not collect information regarding pregnancy planning, so the analysis included eight centers distributed in the five regions. The study protocol was approved by the National Research Ethics Committee – Conep (CAAE No. 31190120.6.1001.5505) – and by each Research Ethics Committee at the place where the data were collected. All participants signed an informed consent form.

Data were collected from June 1, 2020 to August 31, 2020, and enrollment took place over 60 consecutive days at each center. Women who gave birth in university hospitals located in the cities of Manaus, Campo Grande, Brasília, Porto Alegre, Florianópolis, Campinas, São Paulo and Teresina were recruited to participate in the study. Each university hospital had a local coordinator and trained medical residents who participated in data collection.

Inclusion criteria were: age greater than 18 years, single delivery after 36 weeks, live birth without malformations, absence of psychiatric or mental illness in the mother and good maternal health status after delivery. Eligible patients were interviewed in a calm environment on the first or second postpartum day.

### Assessment Instrument

During the preparation of the questionnaire, it was considered that the item should be able to identify a pregnancy that was not the result of a conscious decision by the woman or couple (DeCS/Mesh code G08.686.784.769.570: Unplanned pregnancy^8^ ) regardless of whether or not it was desired at the time of the interview. Therefore, the question presented in the questionnaire was: “Was your pregnancy planned?” and it was followed by two answer options: “Yes, I wanted to have a child this year” or “No, I did not intend to have a child this year”.

### Statistical Analysis

Categorical variables were presented as frequency and continuous variables as median and interquartile range, as they had non-normal distribution, determined by the D’Agostino & Pearson test. The presentation of variables considered the total number of women included in the study, attended to at the eight university hospitals. The unplanned pregnancy rate was also presented considering each university hospital included and each Brazilian region.

Comparison between data related to continuous variables of women with planned and unplanned pregnancies, considering the total sample of women included, was performed using the Mann-Whitney tests. For categorical variables, the odds ratio and the respective 95% confidence interval were calculated using binary logistic regression and the proportions between categories compared using Fisher’s exact test or chi-square. For statistical significance, *p* value < 0.005 was considered. Statistical analysis was performed using the Stata 16.0 program.

## RESULTS

The sample consisted of 1,120 women, whose demographic and obstetric characteristics can be seen in Table 1 .

**Table 1:** Demographic and obstetric characteristics of the sample (n = 1,120).

Characteristics	Results
Age in years – median (IR)	27 (23–32)
Marital status – %	
With partner	86.9
No partner	13.1
Skin color (self-reported) – %	
Black	11.9
White	29.1
Brown	57.2
Yellow	1.2
Indigenous	0.2
Other	0.4
Completed education – %	
None	0.8
Elementary School	22.5
High school	60
College	16.7
Monthly family income, in minimum wages %^a^	
Up to 2	72.7
More than 2	27.3
Number of people living in the same household – % ^b^	
Up to 4	69.2
5 or more	30.8
Religion – %	
Catholic	38.2
Evangelical	33.6
Spiritism	1.3
Other	5.3
None	21.6
Prenatal care	97.8
Number of previous births – %	
0	39.6
1	33
2	15.2
3	8.2
4 or more	4
Previous abortion – %	
Yes	22.6
No	77.4
Number of previous living children – %	
0	38.1
1	35.5
2	15.1
3	7.3
4 or more	4

IR: interquartile range.

^a^95 women did not answer. Calculation made with n = 1,025.

^b^4 women did not answer. Calculation made with n = 1,116.

Of the women interviewed, 756 (67.5%) stated that the pregnancy had not been planned. The median prevalence of UP in the eight participating hospitals was 59.7%. The prevalence of UP was significantly different as the centers were compared with each other (p < 0.0001) ( Table 2 ).

**Table 2:** Prevalence of unplanned pregnancies in eight university hospitals distributed in the five regions of Brazil, from June 1 to August 31, 2020 (n = 1,120).

Region	City	Unplanned pregnancy in each hospital (%)^a^
North	Manaus	95.3
Midwest	Campo Grande	73.9
	Brasília	64.3
Northeast	Teresina	61.2
South	Porto Alegre	58.2
	Florianópolis	59
Southeast	Campinas	54.8
	São Paulo	64.6

^a^ p < 0.0001 (chi-square) when comparing the eight cities.

The median age of women with an unplanned pregnancy (27 years, IR 21–32) was lower than that of those who had planned pregnancy (29 years, IR 24–33) (p = 0.0001) ( Table 3 ).

**Table 3:** Odds ratio of unplanned pregnancy according to sociodemographic and obstetric factors (n = 1,120).

Characteristics	Unplanned pregnancy	Planned pregnancy	OR (CI95%)
	
	(n = 756)	(n = 364)	
Age in years – median (IR)	27 (22-32)	29 (24-33)	
Self-reported color – %			
Not black	28.6	35.7	1 (reference)
Black	71.4	64.3	1.39 (1.06–1.81)
Completed education – %			
None	0.5	1.4	1 (reference)
Elementary School	23	21.4	2.78 (0.73–10.67)
High school	60.3	59.3	2.63 (0.70–9.93)
College	16.1	17.9	2.34 (0.61–9.04)
Monthly family income in minimum wages – %			
Up to 2	75.9	66.1	1 (reference)
More than 2	24.1	33.9	0.62 (0.47–0.83)
Number of people cohabiting at home – %			
1 to 4	65.2	77.5	1 (reference)
5 or more	34.8	22.5	1.84 (1.37–4.91)
Marital status – %			
No partner	17.5	4.1	1 (reference)
With partner	82.5	95.9	0.20 (0.12–0.35)
Religion – %			
Catholic	37	40.7	1 (reference)
Spiritism	1.5	1.1	1.05 (0.78–1.41)
Evangelical	33.1	34.6	1.45 (0.45–4.64)
Other	4.9	6.0	0.89 (1.04–2.08)
None	23.5	17.6	1.47 (1.04–2.08)
Smoking	4.2	5	0.85 (0.47–1.53)
Alcohol use	5.3	4.7	1.14 (0.64–1.04)
Illegal drug use	0.8	1.1	0.72 (0.20–2.57)
Prenatal care – %	97.8	97.8	0.98 (0.42–2.29)
Parity – %			
0	38	43.1	1 (reference)
1	31.5	36.3	0.99 (0.74–1.32)
2	16.4	12.9	1.44 (0.98–2.13)
3	9.1	5.8	1.80 (1.06–3.04)
4 or more	5	1.9	2.97 (1.30–6.81)
Parity - %			
0	38	43.1	1 (reference)
1 or more	62	56.9	1.24 (0.96–1.6)
Previous abortion – %	21.7	24.4	0.86 (0.64–1.15)
Number of living children – %			
0	36.1	42.3	1 (reference)
1	33.9	38.7	1.02 (0.77–1.36)
2	16.4	12.4	1.55 (1.05–2.31)
3	8.6	4.7	2.15 (1.22–3.81)
4 or more	5	1.9	3.06 (1.36–7.02)

IR: interquartile range; OR: odds ratio.

^a^ Man-Whitney test.

^b^ Fisher’s exact test.

^c^ Chi-square test.

Among the epidemiological factors, a greater probability of UP was observed in black women and in those who lived in households with more than four people, while the probability was lower in women who had a partner and in those who had a family income greater than two minimum wages. ( Table 3 ). With regard to obstetric factors, women who had two or more children were more likely to report an unplanned pregnancy ( Table 3 ).

In the studied sample, schooling, religion, history of abortion and consumption of tobacco, alcohol or illicit drugs were not significantly associated with the occurrence of UP ( Table 3 ).

## DISCUSSION

Two-thirds of the women who participated in this study had not planned to become pregnant. Subsequently, to minimize the effect of extreme values, such as that observed in Manaus (95.3%), the median prevalence of the eight participating centers was obtained, whose value was 59.7%. This information can contribute to the understanding of the UP problem in Brazil, but, considering the convenience sampling, restricted to a small group of university hospitals, the results cannot be generalized.

It is currently not possible to compare the prevalence of UP reported in different countries, as there are important methodological differences between studies. Surveys carried out in the last ten years in the United States and Great Britain showed a UP prevalence of around 45%^9 , 10^ , while those carried out in countries in the African continent revealed a mean prevalence of 34% (ranging from 7.5 to 91%) and those in the Asian continent 20% (ranging from 12 to 28%)^11 , 12^ . Among the most relevant methodological differences, the criteria used to define pregnancy as unplanned and the time of pregnancy or puerperium in which the women were interviewed stand out. While in some studies all pregnancies that were not the result of a couple’s conscious decision are considered unplanned, in others they are classified as untimely, when they occurred earlier than desired, and as unwanted, when the woman did not want to become a mother at any time^11^ . With regard to the time of the interview, most studies were based on information obtained from women who had just given birth, so they did not consider pregnancies that ended in miscarriage or abortion in the first half of pregnancy. This may underestimate the prevalence of UP, as it is estimated that between 2010 and 2014 more than half of the UP that occurred in the world ended in abortion^11^ .

In view of this difficulty in comparing studies, longitudinal comparative analyses are essential to evaluate or adjust health policies, as evidenced in the study that showed that the United States reduced the UP rate from 51% to 45% between 2008 and 2011, a change that coincides with the increase in the use of contraceptive methods in all social strata, especially long-term ones, such as the intrauterine device, whose use rate increased from 4% to 12%^8^ .

The only Brazilian study that we are aware of that carried out a longitudinal comparison took place in the municipality of Pelotas, in the southern region, and evaluated the prevalence of UP in 1993, 2004 and 2015^13^ . There was a prevalence of 63% and 66% of UP in the first two periods, falling to 52% in 2015. The change coincided with the record of a lower proportion of families earning less than the minimum wage, a higher proportion of mothers working outside the home, higher maternal educational level and lower proportion of women with two or more children, in addition to a reduction in teenage pregnancy and a higher proportion of mothers aged 30 years or older^13^ .

The finding of younger age in women with UP found in this sample had already been observed in previous studies, pointing to an especially high prevalence among adolescents and women younger than 20 years^3 , 5 , 9 , 12 , 14^ . Although this study only included women aged 18 years or over, which may have underestimated the actual prevalence of UP, it seems clear that younger women are at greater risk and therefore should be the main focus of sexual and reproductive education programs. With regard to marital status, previous studies indicate that the absence of a partner or his negative reaction to the pregnancy are more common in women with unplanned pregnancies, which is consistent with the findings described here^2 , 4 , 5 , 10 , 13^ .

The association between skin color and the risk of UP in Brazil had also been previously pointed out, indicating that women with black, brown or yellow skin have a higher proportion of UP than women with white skin^2 , 5 , 13^ . Likewise, multiparity, more people at home and lower family income, which were associated with UP in this study, reinforce the socially vulnerable profile of this group, as these factors were already evident in studies from the past decade^2 , 5^ . The continuity of the association between social vulnerability and UP is evident in the comparative study carried out in Pelotas, where a drop in the rate of UP from 66% to 52% between 2004 and 2015 was observed, except for the group of women under 24 years old, with more than two children, low educational level, income below the minimum wage and lack of a partner^13^ . A similar phenomenon occurred in the American comparative longitudinal study, in which, despite a drop in the UP rate in all social strata between 2008 and 2011, the rates remained higher in black, poor and Hispanic women, as compared to white women with higher incomes^9^ .

Other factors, such as low education and tobacco, alcohol or illicit drug use, were associated with a higher probability of UP in previous studies, but this association was not observed in our study^2 , 5 , 10^ . As regards education, it is noteworthy that 60% of the women in the sample had completed high school and 16.7% had completed higher education. If this observation is confirmed in further studies, it is worth reflecting on how school curricula contemplate aspects related to sexual and reproductive health. The lack of association between smoking, alcohol and illicit drug use can be ascribed to the fact that, in the present study, the question regarding exposure did not discriminate frequency or volume of exposure, which may have led to the inclusion of women with only occasional consumption as users.

The data presented here are derived from a multicenter cross-sectional study aimed at assessing the emotional impact of the covid-19 pandemic at the end of pregnancy and were collected from women who gave birth between June and August 2020^7^ . Therefore, considering that the pandemic was declared on March 11 of that year, it is important to emphasize that the women interviewed became pregnant before the start of the health emergency and therefore their responses regarding the pregnancy schedule were not influenced by this situation.

Although conducting a nationwide survey was an opportunity to explore the prevalence of UP in hospitals in all regions of Brazil, the fact that the study was not specifically designed for this purpose imposed several limitations on it. Convenience sampling, restricted to university hospitals, which aimed to improve the quality of data collection, certainly selected a sample that is not representative of the universe of Brazilian pregnant women and, therefore, the data presented here cannot be generalized to the general population.

As in previous Brazilian studies, the fact that only women who had just given birth were questioned did not allow us to know the proportion of UP that ended in abortion. It is estimated that, globally, 61% of UP ends in abortion and that the percentage is higher in developing countries (66%) than in highly developed ones (43%)^1^ . In the study that compared the prevalence of UP in the United States in 2008 and 2011, stability was observed in the proportion of UP culminating in abortion in these two periods, around 40%^9^ . Likewise, it is estimated that in France 38% of UP culminate in abortions^16^ . Therefore, when interviewing only women who maintained their pregnancies, it is possible that the prevalence of UP was underestimated.

Another factor that compromises the reliability of the prevalence found is that the question regarding pregnancy planning was binary, so that it did not consider ambivalent feelings or the gradation of intentionality/pregnancy planning. Specifically, when asking the researchers who collected the data in Manaus about the very high prevalence of UP found, they reported the impression that the binary response option did not allow assessing the cultural lack of concern about the number and time of arrival of children, characteristic of the public served in this hospital.

Finally, the fact of excluding adolescent women and women with psychiatric illnesses may have underestimated the prevalence of UP, since it is well established that in adolescence there is a greater probability of accidental pregnancy and that UP is a risk factor for depressive conditions during pregnancy^3 , 5 , 9 , 10 , 15 , 17^ .

Considering that the choice of the number of children and the moment to have them are reproductive rights that must be guaranteed to every human being and that the real prevalence of UP in Brazil remains unknown, the need for studies designed specifically for this purpose and using instruments created for that purpose is clear. Recently, the Brazilian Portuguese version of the London Measure of Unplanned Pregnancy (LMUP) was validated, a self-administered six-question scale that results in a score from 0 to 12 points concerning the intentionality/planning of pregnancy, where 0 to 3 characterizes it as unplanned, 4 to 9 as ambivalent, and 10 to 12 as planned. Hopefully, future studies with the application of this instrument in representative samples of the Brazilian population will bring to light more reliable and comparable information.
