# Supplementary material for: Unplanned pregnancy in Brazil: national study in eight university hospitals
Source: Rev Saude Publica. 2023 Jun 2;57:35. doi: 10.11606/s1518-8787.2023057004449 (PMC10317132; doi:10.11606/s1518-8787.2023057004449)
Supplement: Translation

# Translation: Gravidez não planejada no Brasil: estudo nacional em oito hospitais universitários

**Authors:** Tainá Vieira Nilson, Angélica Amorim Amato, Ceres Nunes Resende, Walquíria Quida Salles Pereira Primo, Roseli Mieko Yamamoto Nomura, Maria Laura Costa, Maria Lúcia Opperman, Marianna Brock, Alberto Trapani, Lia Cruz Vaz da Costa Damasio, Nadia Reis, Vera Borges, Ana Cristina Araújo, Rodrigo Ruano, Alberto Carlos Moreno Zaconeta

**Affiliations:** I Brasil Universidade de Brasília. Hospital Universitário de Brasília. Brasília, DF, Brasil; II Brasil Universidade de Brasília. Faculdade de Ciências da Saúde. Departamento de Ciências Farmacêuticas. Brasília, DF, Brasil; III Brasil Universidade de Brasília. Faculdade de Medicina. Área de Ginecologia e Obstetrícia. Brasília, DF, Brasil; IV Brasil Universidade Federal de São Paulo. Escola Paulista de Medicina. Departamento de Obstetrícia. São Paulo, SP, Brasil; V Brasil Universidade Estadual de Campinas. Departamento de Ginecologia e Obstetrícia. Campinas, SP, Brasil; VI Brasil Universidade Federal do Rio Grande do Sul. Faculdade de Medicina. Unidade de Ginecologia e Obstetrícia. Porto Alegre, RS, Brasil; VII Brasil Universidade Federal do Amazonas. Departamento de Obstetrícia e Ginecologia. Manaus, AM, Brasil; VIII Brasil Universidade Federal de Santa Catarina. Hospital Universitário Polydoro Ernani de São Thiago. Unidade de Saúde da Mulher. Florianópolis, SC, Brasil; IX Brasil Universidade Federal do Piauí. Departamento de Ginecologia e Obstetrícia. Teresina, PI, Brasil; X Brasil Universidade Federal de Mato Grosso do Sul. Hospital Universitário Maria Aparecida Pedrossian. Unidade de Ginecologia e Obstetrícia. Campo Grande, MS, Brasil; XI Brasil Universidade Estadual Paulista. Escola de Medicina de Botucatu. Departamento de Ginecologia e Obstetrícia. Botucatu, SP, Brasil; XII Brasil Universidade Federal do Rio Grande do Norte. Maternidade Januário Cicco. Departamento de Ginecologia e Obstetrícia. Natal, RN, Brasil; XIII USA Mayo Clinic College of Medicine, Department of Obstetrics and Gynecology. Maternal-Fetal Medicine Division. Rochester, USA

**Keywords:** Gravidez Não Planejada, Planejamento Familiar, Anticoncepção, Direitos Sexuais e Reprodutivos

## Abstract

**OBJETIVO:**

Estimar a prevalência de gestação não planejada (GNP) em oito hospitais públicos universitários, distribuídos nas cinco regiões que compõem o Brasil.

**MÉTODOS:**

Análise secundária de um estudo transversal multicêntrico nacional, realizado em oito hospitais universitários públicos, entre 1º de junho e 31 de agosto de 2020, no Brasil. Amostra por conveniência incluindo mulheres que deram à luz em período de sessenta dias consecutivos e atenderam aos seguintes critérios: maiores de 18 anos; idade gestacional acima de 36 semanas no parto; com recém-nascido único e vivo, sem malformações.

**RESULTADOS:**

Amostra composta por 1.120 puérperas, das quais 756 (67,5%) declararam que a gravidez não tinha sido programada. A mediana da prevalência de GNP foi de 59,7%. Observou-se diferença significativa na prevalência de GNP entre os hospitais: Campinas (54,8%), Porto Alegre (58,2%), Florianópolis (59%), Teresina (61,2%), Brasília (64,3%), São Paulo (64,6%), Campo Grande (73,9%) e Manaus (95,3%) (p < 0,001). Foram fatores significativamente associados a GNP a idade materna, cor negra, menor renda familiar, maior número de filhos, maior número de pessoas convivendo em casa e não ter parceiro.

**CONCLUSÃO:**

Na amostra estudada, cerca de dois terços das gestações foram declaradas como não programadas. A prevalência de gestação não planejada teve relação com fatores sociais e demográficos e variou significativamente entre os hospitais universitários avaliados.

## INTRODUÇÃO

A redução do tamanho das famílias decorrente do adiamento da parentalidade e das menores taxas de natalidade é uma tendência global e sugere maior acesso a métodos contraceptivos eficientes^1^ . No entanto, dados recentes apontam que 48% das gestações ocorridas no mundo nos últimos cinco anos não foram planejadas, o que representa 121 milhões de casos por ano ou uma taxa anual global de 64 gestações não planejadas (GNP) para cada mil mulheres entre 15 e 49 anos^1^ . A taxa anual de gestações não planejadas a cada mil mulheres em idade reprodutiva varia de forma inversamente proporcional ao grau de desenvolvimento socioeconômico de cada país, sendo de 34 em países desenvolvidos, 66 em países com índice médio de desenvolvimento e 93 naqueles com baixo índice^1^ . Dessa forma, os países com baixo índice de desenvolvimento concentram as maiores taxas de GNP.

No Brasil, um inquérito realizado na região Sul investigou todos os partos ocorridos em um município ao longo de 2007 e encontrou uma taxa de gestação não planejada de 65% nas 2.500 mulheres entrevistadas^2^ . Resultado semelhante foi descrito em um estudo realizado em 2010, que avaliou os dados de mais de cinco mil mulheres em uma capital da região Nordeste e encontrou prevalência de GNP de 68%^3^ . Nesse mesmo ano, um questionário aplicado em todas as maternidades do município de Ribeirão Preto, na região Sudeste do país, encontrou prevalência de GNP de 54% nas 7.500 mulheres entrevistadas^4^ . De maior envergadura, o estudo “Nascer no Brasil”, que avaliou 24 mil mulheres que tiveram parto entre 2011 e 2012, mostrou que 55% das gestações não tinham sido planejadas e que havia diferenças importantes no acesso à saúde perinatal nas distintas regiões do Brasil^4^ . Os dados de todos esses estudos indicaram de forma convergente que o grupo de mulheres com gestações não planejadas apresentava alto grau de vulnerabilidade social^2 , 4-6^ .

Transcorrida uma década desde o último inquérito, tivemos a oportunidade de explorar a prevalência de gestações não planejadas em maternidades das distintas regiões do país. O objetivo do presente estudo foi determinar a frequência de GNP nas maternidades de oito hospitais públicos universitários distribuídos ao longo das cinco regiões geográficas do Brasil.

## MÉTODOS

### Desenho do Estudo e Composição da Amostra

Esta é uma análise de dados secundária de um estudo maior,^7^ cujo desenho transversal multicêntrico permitiu a coleta de dados em cidades das cinco regiões do Brasil (Norte, Nordeste, Centro-Oeste, Sudeste e Sul). Dos dez centros que participaram do estudo maior, dois não levantaram informações quanto ao planejamento da gestação, de forma que a análise incluiu oito centros distribuídos nas cinco regiões. O protocolo do estudo foi aprovado pelo Comitê Nacional de Ética em Pesquisa – Conep (CAAE nº 31190120.6.1001.5505) – e por cada comitê de ética em Pesquisa do local onde os dados foram coletados. Todas as participantes assinaram um termo de consentimento informado.

Os dados foram coletados no período de 1º de junho de 2020 a 31 de agosto de 2020 e as inscrições ocorreram durante 60 dias consecutivos em cada centro. As mulheres que tiveram parto em hospitais universitários localizados nas cidades de Manaus, Campo Grande, Brasília, Porto Alegre, Florianópolis, Campinas, São Paulo e Teresina foram recrutadas para participar do estudo. Cada hospital universitário tinha um coordenador local e residentes médicos treinados, que participaram da coleta de dados.

Os critérios de inclusão foram: idade maior que 18 anos, parto único após 36 semanas, recém-nascido vivo sem malformações, ausência de doença psiquiátrica ou mental na mãe e bom estado de saúde materna após o parto. As pacientes elegíveis foram entrevistadas em um ambiente calmo no primeiro ou segundo dia pós-parto.

### Instrumento de Avaliação

Durante a elaboração do questionário, ponderou-se que o item deveria ser capaz de identificar a gravidez que não resultou de decisão consciente da mulher ou casal (código DeCS/Mesh G08.686.784.769.570: Gravidez não planejada^8^ ) independentemente de ser ou não desejada no momento da entrevista. Para tanto, no questionário foi apresentada a pergunta “A sua gravidez foi programada?”, seguida de duas opções de resposta: “Sim, eu queria ter um filho este ano” ou “Não, eu não pretendia ter um filho este ano”.

### Análise Estatística

As variáveis categóricas foram apresentadas como frequência e as variáveis contínuas como mediana e intervalo interquartil, pois apresentaram distribuição não normal, determinada pelo teste de D’Agostino & Pearson. A apresentação das variáveis considerou o total de mulheres incluídas no estudo, atendidas nos oito hospitais universitários. A taxa de gestação não programada foi também apresentada considerando cada hospital universitário incluído e cada região brasileira.

A comparação entre os dados relativos às variáveis contínuas de mulheres com gestação planejada e não planejada, considerando a amostra total de mulheres incluídas, foi feita através dos testes de Mann-Whitney. Para as variáveis categóricas, foi calculada a razão de chances e o respectivo intervalo de confiança de 95% por meio de regressão logística binária e as proporções entre as categorias comparadas por meio do teste exato de Fisher ou qui-quadrado. Para significância estatística foi considerado o valor de p < 0,005. A análise estatística foi realizada com o programa Stata 16.0.

## RESULTADOS

A amostra foi composta por 1.120 mulheres, cujas características demográficas e obstétricas podem ser vistas na Tabela 1 .

**Tabela 1:** Características demográficas e obstétricas da amostra (n = 1.120).

Características	Resultados
Idade em anos – mediana (IIQ)	27 (23–32)
Estado conjugal – %	
Com companheiro	86,9
Sem companheiro	13,1
Cor da pele (autorreferida) – %	
Negra	11,9
Branca	29,1
Parda	57,2
Amarela	1,2
Indígena	0,2
Outra	0,4
Escolaridade completa – %	
Nenhuma	0,8
Ensino fundamental	22,5
Ensino médio	60
Ensino superior	16,7
Renda familiar mensal, em salários mínimos %^a^	
Até 2	72,7
Mais de 2	27,3
Número de pessoas que habitam a mesma casa – %^b^	
Até 4	69,2
5 ou mais	30,8
Religião %	
Católica	38,2
Evangélica	33,6
Espírita	1,3
Outra	5,3
Nenhuma	21,6
Assistência pré-natal	97,8
Número de partos anteriores – %	
0	39,6
1	33
2	15,2
3	8,2
4 ou mais	4
Abortos anteriores – %	
Sim	22,6
Não	77,4
Número de filhos vivos anteriores – %	
0	38,1
1	35,5
2	15,1
3	7,3
4 ou mais	4

IIQ: intervalo interquartil.

^a^95 mulheres não responderam. Cálculo feito com n = 1.025.

^b^4 mulheres não responderam. Cálculo feito com n = 1.116.

Das mulheres entrevistadas, 756 (67,5%) declararam que a gravidez não tinha sido planejada. A mediana da prevalência de GNP nos oito hospitais participantes foi de 59,7%. A prevalência de GNP foi significativamente diferente ao comparar os centros entre si (p < 0,0001) ( Tabela 2 ).

**Tabela 2:** Prevalência de gravidez não planejada em oito hospitais universitários distribuídos nas cinco regiões do Brasil, no período de 1º de junho a 31de agosto de 2020 (n = 1.120).

Região	Cidade	Gestação não planejada em cada hospital (%)^a^
Norte	Manaus	95,3
Centro-Oeste	Campo Grande	73,9
	Brasília	64,3
Nordeste	Teresina	61,2
Sul	Porto Alegre	58,2
	Florianópolis	59
Sudeste	Campinas	54,8
	São Paulo	64,6

^a^p < 0,0001 (qui-quadrado) ao comparar as oito cidades.

A mediana da idade das mulheres com gestação não planejada (27 anos, IIQ 21–32) foi menor que a daquelas que tinham planejado a gravidez (29 anos, IIQ 24–33) (p = 0,0001) ( Tabela 3 ).

**Tabela 3:** Razão de chances de gestação não planejada de acordo com fatores sociodemográficos e obstétricos (n = 1.120).

Características	Gravidez não planejada	Gravidez planejada	OR (IC95%)
	
	(n = 756)	(n = 364)	
Idade em anos – mediana (IIQ)	27 (22-32)	29 (24-33)	
Cor autodeclarada – %			
Não negra	28,6	35,7	1 (referência)
Negra	71,4	64,3	1,39 (1,06–1,81)
Escolaridade completa – %			
Nenhuma	0,5	1,4	1 (referência)
Ensino Fundamental	23	21,4	2,78 (0,73–10,67)
Ensino Médio	60,3	59,3	2,63 (0,70–9,93)
Ensino Superior	16,1	17,9	2,34 (0,61–9,04)
Renda familiar mensal em salários-mínimos – %			
Até 2	75,9	66,1	1 (referência)
Mais de 2	24,1	33,9	0,62 (0,47–0,83)
Número de pessoas coabitando em casa – %			
1 a 4	65,2	77,5	1 (referência)
5 ou mais	34,8	22,5	1,84 (1,37–4,91)
Situação conjugal – %			
Sem companheiro	17,5	4,1	1 (referência)
Com companheiro	82,5	95,9	0,20 (0,12–0,35)
Religião – %			
Católica	37	40,7	1 (referência)
Espírita	1,5	1,1	1,05 (0,78–1,41)
Evangélica	33,1	34,6	1,45 (0,45–4,64)
Outra	4,9	6,0	0,89 (1,04–2,08)
Nenhuma	23,5	17,6	1,47 (1,04–2,08)
Tabagismo	4,2	5	0,85 (0,47–1,53)
Uso de álcool	5,3	4,7	1,14 (0,64–1,04)
Uso de drogas ilícitas	0,8	1,1	0,72 (0,20–2,57)
Atenção pré-natal – %	97,8	97,8	0,98 (0,42–2,29)
Paridade – %			
0	38	43,1	1 (referência)
1	31,5	36,3	0,99 (0,74–1,32)
2	16,4	12,9	1,44 (0,98–2,13)
3	9,1	5,8	1,80 (1,06–3,04)
4 ou mais	5	1,9	2,97 (1,30–6,81)
Paridade – %			
0	38	43,1	1 (referência)
1 ou mais	62	56,9	1,24 (0,96–1,6)
Aborto anterior – %	21,7	24,4	0,86 (0,64–1,15)
Número de filhos vivos – %			
0	36,1	42,3	1 (referência)
1	33,9	38,7	1,02 (0,77–1,36)
2	16,4	12,4	1,55 (1,05–2,31)
3	8,6	4,7	2,15 (1,22–3,81)
4 ou mais	5	1,9	3,06 (1,36–7,02)

IIQ: intervalo interquartil; OR: razão de chances ( *odds ratio* ).

^a^Teste Man-Whitney.

^b^Teste exato de Fisher.

^c^Teste qui-quadrado.

Entre os fatores epidemiológicos, observou-se maior probabilidade de GNP em mulheres negras e naquelas que viviam em casas com número de pessoas superior a quatro, enquanto a probabilidade foi menor nas mulheres que tinham parceiro e nas que tinham renda familiar maior que dois salários mínimos ( Tabela 3 ). No que diz respeito aos fatores obstétricos, observou-se que mulheres que tinham dois ou mais filhos tinham maior probabilidade de declarar a gestação como não planejada ( Tabela 3 ).

Na amostra estudada, escolaridade, religião, antecedente de aborto e consumo de tabaco, álcool ou drogas ilícitas não tiveram associação significativa com a ocorrência de GNP ( Tabela 3 ).

## DISCUSSÃO

Dois terços das mulheres que participaram deste estudo não haviam planejado engravidar. Posteriormente, para minimizar o efeito de valores extremos, como o observado em Manaus (95,3%), foi obtida a mediana da prevalência dos oito centros participantes, cujo valor foi 59,7%. Essa informação pode contribuir para a compreensão do problema da GNP no Brasil, mas, considerando a amostragem por conveniência, restrita a um pequeno grupo de hospitais universitários, os resultados não podem ser generalizados.

Atualmente não é possível comparar a prevalência de GNP reportada em diversos países, pois há importantes diferenças metodológicas entre os estudos. Inquéritos realizados nos últimos dez anos nos Estados Unidos e na Grã-Bretanha mostraram prevalência de GNP em torno de 45%^9 , 10^ , enquanto os realizados em países do continente africano revelaram prevalência média de 34% (variação de 7,5 a 91%) e os do continente asiático 20% (variação de 12 a 28%)^11 , 12^ . Dentre as diferenças metodológicas mais relevantes destacam-se o critério usado para definir a gravidez como não planejada e o momento da gravidez ou puerpério em que as mulheres foram entrevistadas. Enquanto em alguns estudos considera-se não planejada toda gravidez que não decorreu de decisão consciente do casal, em outros essas são classificadas em inoportunas, quando ocorreram antes do desejado, e indesejadas, quando a mulher não queria se tornar mãe em momento algum^11^ . No que diz respeito ao momento da entrevista, a maior parte dos estudos se basearam em informações obtidas em mulheres que acabaram de ter um parto, de forma que não consideraram as gravidezes que terminaram em aborto espontâneo ou intencional na primeira metade da gravidez. Isso pode subestimar a prevalência de GNP, pois se estima que entre 2010 e 2014 mais da metade das GNP ocorridas no mundo terminaram em aborto^11^ .

Em face dessa dificuldade de comparação entre estudos, análises comparativas longitudinais são essenciais para avaliar ou ajustar as políticas de saúde, como ficou evidente no estudo que mostrou que os Estados Unidos reduziram a taxa de GNP de 51% para 45% entre os anos de 2008 e 2011, mudança coincidente com o aumento do uso de métodos contraceptivos em todos os estratos sociais, principalmente os de longa duração, como o dispositivo intrauterino, cuja taxa de uso passou de 4% para 12%^8^ .

O único estudo brasileiro de que temos conhecimento que fez uma comparação longitudinal ocorreu no município de Pelotas, na região Sul, e avaliou a prevalência de GNP nos anos de 1993, 2004 e 2015^13^ . Observou-se prevalência de 63 e 66% de GNP nos dois primeiros períodos, com queda para 52% em 2015. A mudança coincidiu com o registro de menor proporção de famílias recebendo menos de um salário mínimo, maior proporção de mães trabalhando fora do lar, maior nível educacional materno e menor proporção de mulheres com dois ou mais filhos, além de redução de gestação na adolescência e maior proporção de mães com idade igual ou superior a 30 anos^13^ .

O achado de menor idade em mulheres com GNP encontrado nesta amostra já tinha sido observado em estudos prévios, apontando prevalência especialmente alta entre adolescentes e mulheres menores de 20 anos^3 , 5 , 9 , 12 , 14^ . Apesar deste estudo ter incluído apenas mulheres com idade igual ou superior a 18 anos, o que pode ter subestimado a real prevalência de GNP, parece claro que as mulheres mais jovens têm mais risco e portanto devem ser foco principal dos programas de educação sexual e reprodutiva. No que diz respeito ao estado conjugal, estudos anteriores indicam que a ausência de parceiro ou a reação negativa deste em relação à gestação são mais comuns em mulheres com gestações não planejadas, o que é concordante com o resultado aqui descrito^2 , 4 , 5 , 10 , 13^ .

A associação entre a cor da pele e o risco de GNP no Brasil também já tinha sido previamente apontada, indicando de forma convergente que mulheres com pele preta, parda ou amarela apresentam maior proporção de GNP que mulheres com pele branca^2 , 5 , 13^ . Igualmente, multiparidade, maior número de pessoas em casa e menor renda familiar, que foram associados a GNP neste estudo, reforçam o perfil socialmente vulnerável desse grupo, pois esses fatores já eram evidentes nos estudos da década passada^2 , 5^ . A perenidade da associação entre vulnerabilidade social e GNP fica patente no estudo comparativo realizado em Pelotas, onde se observou queda da taxa de GNP de 66% para 52% entre 2004 e 2015, exceto para o grupo de mulheres com menos de 24 anos, com mais de dois filhos, baixo nível educacional, renda inferior a um salário mínimo e ausência de parceiro^13^ . Fenômeno semelhante ocorreu no estudo longitudinal comparativo americano, no qual, apesar de ter ocorrido queda da taxa de GNP em todos os estratos sociais entre 2008 e 2011, os índices permaneceram superiores em mulheres negras, pobres e hispânicas, quando comparadas a brancas de maior renda^9^ .

Outros fatores, como baixa escolaridade e uso de tabaco, álcool ou drogas ilícitas, foram associados a maior probabilidade de GNP em estudos pregressos, mas essa associação não foi observada em nosso estudo^2 , 5 , 10^ . No que diz respeito à escolaridade, chama a atenção que 60% das mulheres que compuseram a amostra tinha concluído o ensino médio e 16,7% o ensino superior. Se essa observação for confirmada em estudos posteriores, cabe a reflexão sobre a forma como os currículos escolares contemplam aspectos relacionados à saúde sexual e reprodutiva. A falta de associação entre tabagismo, uso de álcool e drogas ilícitas pode ser explicada porque no presente estudo a pergunta quanto à exposição não discriminava frequência ou volume da exposição, o que pode ter levado a incluir como usuárias mulheres com consumo apenas ocasional.

Os dados aqui apresentados são derivados de um estudo transversal multicêntrico destinado a avaliar o impacto emocional da pandemia de covid-19 no final da gestação e foram colhidos em mulheres que tiveram parto entre junho e agosto de 2020^7^ . Portanto, considerando que a pandemia foi declarada em 11 de março desse ano, é importante ressaltar que as mulheres entrevistadas engravidaram antes do início da emergência sanitária e por isso as suas respostas a respeito da programação da gestação não foram influenciadas por essa situação.

Apesar da realização de um inquérito de abrangência nacional ter sido uma oportunidade de explorar a prevalência de GNP em hospitais de todas as regiões do Brasil, o fato do estudo não ter sido especificamente desenhado para esse fim lhe impôs várias limitações. A amostragem por conveniência, restrita a hospitais universitários, que teve por objetivo aprimorar a qualidade da coleta de dados, certamente selecionou uma amostra que não é representativa do universo de gestantes brasileiras e, portanto, os dados aqui apresentados não podem ser generalizados para a população.

À semelhança dos estudos brasileiros anteriores, o fato de se ter interrogado apenas mulheres que tinham acabado de ter filho não permitiu conhecer a proporção de GNP terminadas em aborto. Estima-se que, globalmente, 61% das GNP terminam em abortamento intencional e que o percentual é maior em países em desenvolvimento (66%) do que naqueles altamente desenvolvidos (43%)^1^ . No estudo que comparou a prevalência de GNP nos Estados Unidos nos anos 2008 e 2011 observou-se estabilidade da proporção de GNP culminadas em abortamento nesses dois períodos, em torno de 40%^9^ . Igualmente, estima-se que na França 38% das GNP culminam em abortamentos intencionais^16^ . Por tanto, ao entrevistar apenas mulheres que mantiveram a gravidez, é possível que a prevalência de GNP tenha sido subestimada.

Outro fator que compromete a fidedignidade da prevalência encontrada é que a pergunta quanto ao planejamento da gravidez foi binária, de forma que não considerou sentimentos ambivalentes ou a gradação de intencionalidade/planejamento da gestação. Especificamente, ao indagar aos pesquisadores que coletaram os dados em Manaus acerca da altíssima prevalência de GNP encontrada, relataram a impressão de que a opção de resposta binária não permitiu avaliar a despreocupação cultural acerca do número e momento de chegada dos filhos, característica do público atendido nesse hospital.

Finalmente, o fato de ter excluído mulheres adolescentes e com doenças psiquiátricas pode ter subestimado a prevalência de GNP, uma vez que está bem definido que na adolescência há maior probabilidade de gravidez acidental e que a GNP é fator de risco para quadros depressivos durante a gestação^3 , 5 , 9 , 10 , 15 , 17^ .

Considerando que a escolha do número de filhos e do momento de tê-los são direitos reprodutivos que devem ser assegurados a todo ser humano e que a real prevalência de GNP no Brasil permanece desconhecida, fica patente a necessidade de estudos desenhados especificamente para esse fim e utilizando instrumentos criados para esse propósito. Recentemente, foi validada a versão em português brasileiro da *London Measure of Unplanned Pregnancy* (LMUP), escala autoaplicável com seis questões que resulta em um escore de 0 a 12 pontos referentes à intencionalidade/planejamento da gravidez, que caracteriza 0 a 3 como não planejada, 4 a 9 como ambivalente e 10 a 12 como planejada. Espera-se que estudos futuros com a aplicação desse instrumento em amostras representativas da população brasileira tragam à luz informações mais fidedignas e comparáveis.
